# α-Terpineol Attenuates PS-NP-Induced Lung Injury in Association with NF-κB Signaling and GSDMD-Related Pyroptosis

**DOI:** 10.3390/ijms272110045

**Published:** 2026-11-09

**Authors:** Xiaobing Zhang, Xiaoqing Li, Yingling Luo, Jiawen Tang, Pengjing Li, Qi Wang

**Affiliations:** 1 School of Public Health, Kunming Medical University, Kunming 650500, China; 20240271@kmmu.edu.cn (X.Z.);; 2 Experiment Center for Medical Science Research, Kunming Medical University, Kunming 650500, China

**Keywords:** α-Terpineol, polystyrene nanoplastics, lung injury, NF-κB, NLRP3, pyroptosis, GSDMD

## Abstract

Emerging environmental contaminants called polystyrene nanoplastics (PS-NPs) may be harmful to respiratory health. With a focus on inflammatory responses, NF-κB signaling, and GSDMD-mediated pyroptosis, this work examined the impact of α-terpineol (α-TPN), a naturally occurring monoterpene with antioxidant and anti-inflammatory qualities, on PS-NP-induced lung damage. A549 cells were initially used to assess the effects of PS-NPs and α-TPN. CCK-8, LDH release, and Annexin V/PI flow cytometry were used to measure cell viability, cellular damage, and cell death, respectively. ELISA was used to measure the levels of IL-1β, IL-6, and IL-8, while NF-κB signaling and pyroptosis-related proteins were examined by Western blotting and immunofluorescence. The protective effects of α-TPN on lung function and lung pathology were then assessed using a mouse model of PS-NP-induced lung injury. IL-1β, IL-6, and TNF-α levels in bronchoalveolar lavage fluid were measured to evaluate inflammatory responses. NF-κB signaling, NLRP3/ASC inflammasome-associated proteins, and GSDMD-related pyroptosis were further evaluated in lung tissues using Western blotting, immunohistochemistry, and immunofluorescence. PS-NP exposure increased LDH release, Annexin V-positive cell death, and IL-1β, IL-6, and IL-8 levels in A549 cells. These effects were associated with enhanced NF-κB p65 signaling and changes in the expression of pyroptosis-related proteins, including NLRP3, ASC, cleaved caspase-1, and GSDMD-N. α-TPN pretreatment attenuated cellular injury and inflammatory responses and partially reversed these protein changes. In mice, PS-NP exposure impaired pulmonary function, characterized by increased enhanced pause (Penh) and decreased expiratory flow at 50% expired volume (EF50), minute ventilation (MV), and respiratory frequency (fbpm). Alveolar wall thickening, edema, inflammatory cell infiltration, and structural damage were also brought on by PS-NP exposure, and bronchoalveolar lavage fluid (BALF) showed increased levels of TNF-α, IL-1β, and IL-6. Treatment with α-TPN decreased BALF inflammatory cytokine levels and enhanced lung histology and pulmonary function. Additionally, α-TPN decreased GSDMD-N, cleaved caspase-1, mature IL-18, and mature IL-1β levels in lung tissues and inhibited changes in NF-κB-related proteins, indicating that its protective effects may entail modulation of GSDMD-related pyroptosis and NF-κB-associated inflammatory signaling. α-TPN alleviates PS-NP-induced lung injury and inflammatory responses, potentially by regulating inflammation and pyroptosis. These results imply that α-TPN could be a natural active substance for reducing lung damage caused by PS-NPs.

## 1. Introduction

Due to the widespread production and use of plastics, microplastics (MPs) and nanoplastics (NPs) are ubiquitous in the environment and have become pollutants of growing concern [1]. Because of their small size, NPs can enter the respiratory system and travel to the lungs, where they may settle in distal pulmonary areas [2]. An increasing amount of research suggests that lung exposure to nanoplastics may result in inflammation, oxidative stress, and structural lung damage [3]. Nanoplastics have a higher specific surface area and surface reactivity than larger microplastics, which may increase their potential for harmful impacts on respiratory health [4]. Among several forms of nanoplastics, polystyrene nanoplastics (PS-NPs) are commonly employed as model particles in toxicological investigations because of their well-characterized physicochemical features and availability for laboratory study [5]. There is accumulating evidence that PS-NPs can cause oxidative stress, pulmonary inflammation, and lung structural damage. However, little is known about the underlying mechanisms of lung damage caused by PS-NPs and effective therapeutic strategies.

Inflammation and oxidative stress are two important pathogenic processes in lung injury caused by PS-NPs [6]. Following inhalation, PS-NPs may come into contact with pulmonary epithelial and immune cells, trigger ROS production, and stimulate inflammatory signaling pathways. These responses can increase the release of inflammatory mediators and contribute to lung injury [7].

Within these reactions, inflammatory effects and inflammatory programmed cell death caused by PS-NPs may be further enhanced by activation of the NF-κB pathway [8]. The inflammatory kind of programmed cell death known as pyroptosis is linked to the activation of the NLRP3 inflammasome, caspase-1 cleavage, and GSDMD, which in turn stimulates the release of inflammatory cytokines such as IL-1β and IL-18 [9]. According to recent research, exposure to PS-NPs can cause pulmonary pyroptosis along with the activation of inflammatory and pyroptosis-related pathways. This implies that the development of PS-NP-induced lung damage may be facilitated by pyroptosis [10]. Further research is needed to fully understand the regulatory processes that connect PS-NP exposure to pyroptosis, including the functions of inflammatory signaling and GSDMD-mediated pyroptosis.

α-Terpineol (α-TPN) is a naturally occurring monoterpene commonly found in the essential oils of various aromatic plants [11]. Previous studies have shown that α-TPN can lower inflammatory responses and oxidative stress in numerous animal models, with protective effects seen in various types of tissue damage [12,13]. Previous studies have shown that α-TPN can suppress NF-κB-associated inflammatory signaling and reduce the production of pro-inflammatory mediators. Its antioxidant properties may also help limit oxidative stress [14,15]. These findings spurred more investigation into the underlying pyroptotic and inflammatory processes as well as the advantages of α-TPN in preventing lung damage from PS-NPs.

Uncertainty surrounds α-TPN’s preventive benefits against pulmonary damage caused by PS-NPs and its possible involvement in inflammatory cell death. This work examined the effects of α-TPN on PS-NP-induced lung injury utilizing A549 cells and mice models, with an emphasis on inflammation, NF-κB signaling, and GSDMD-mediated pyroptosis.

## 2. Results

### 2.1. Effects of α-Terpineol and PS-NPs on A549 Cell Viability and Cell Death

**Figure 1:**
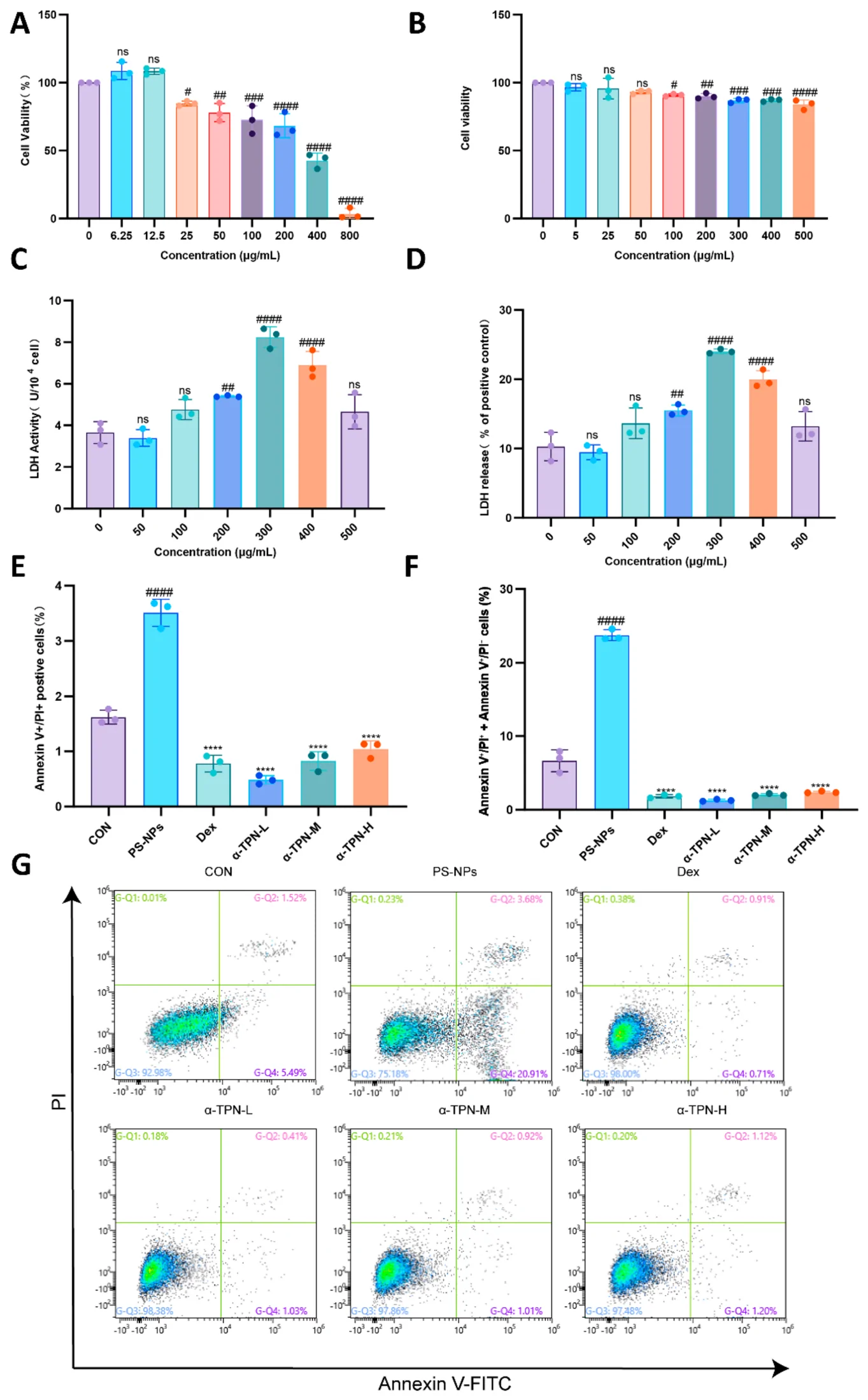
Effects of α-terpineol and PS-NPs on A549 cell viability and cell death. (**A**) Effect of α-TPN at different concentrations on A549 cell viability; (**B**) effect of PS-NPs at different concentrations on A549 cell viability; (**C**) LDH activity in A549 cells exposed to different concentrations of PS-NPs; (**D**) LDH release rate in A549 cells exposed to different concentrations of PS-NPs; (**E**) proportion of Annexin V^+^/PI^+^ cells (Q2); (**F**) total proportion of Annexin V-positive cells, including Annexin V^+^/PI^−^ and Annexin V^+^/PI^+^ cells (Q2 + Q4); (**G**) representative flow cytometry plots of Annexin V-FITC/PI staining. # *p* < 0.05, ## *p* < 0.01, ### *p* < 0.001, and #### *p* < 0.0001 indicate statistically significant differences compared with the Control group; **** *p* < 0.0001 indicate statistically significant differences compared with the PS-NPs group. ns, not significant (*p* ≥ 0.05).

A549 cell viability was not significantly affected by α-TPN at 6.25 and 12.5 μg/mL, whereas 25 μg/mL reduced viability to approximately 85% of the control level (*p* < 0.05) (Figure 1A). The calculated IC50 was 252.3 μg/mL. Accordingly, 6.25, 12.5, and 25 μg/mL were selected for subsequent experiments.

PS-NP exposure (5–500 μg/mL) significantly reduced cell viability at concentrations of 100–500 μg/mL, although viability remained above 80% (Figure 1B). Both LDH activity and release rate increased with PS-NP concentration, peaked at 300 μg/mL, and declined at higher concentrations (Figure 1C,D). Therefore, 300 μg/mL was selected for subsequent experiments.

Flow cytometry further showed that PS-NPs increased the proportions of Annexin V^+^/PI^+^ cells (Q2) and total Annexin V-positive cells (Q2 + Q4) compared with the Control group (both *p* < 0.0001) (Figure 1E,F). These proportions were significantly reduced by DEX and α-TPN pretreatment compared with the PS-NP group (all *p* < 0.0001). Representative flow cytometry plots are presented in Figure 1G.

### 2.2. α-Terpineol Attenuates PS-NP-Induced Inflammatory Responses in A549 Cells

**Figure 2:**
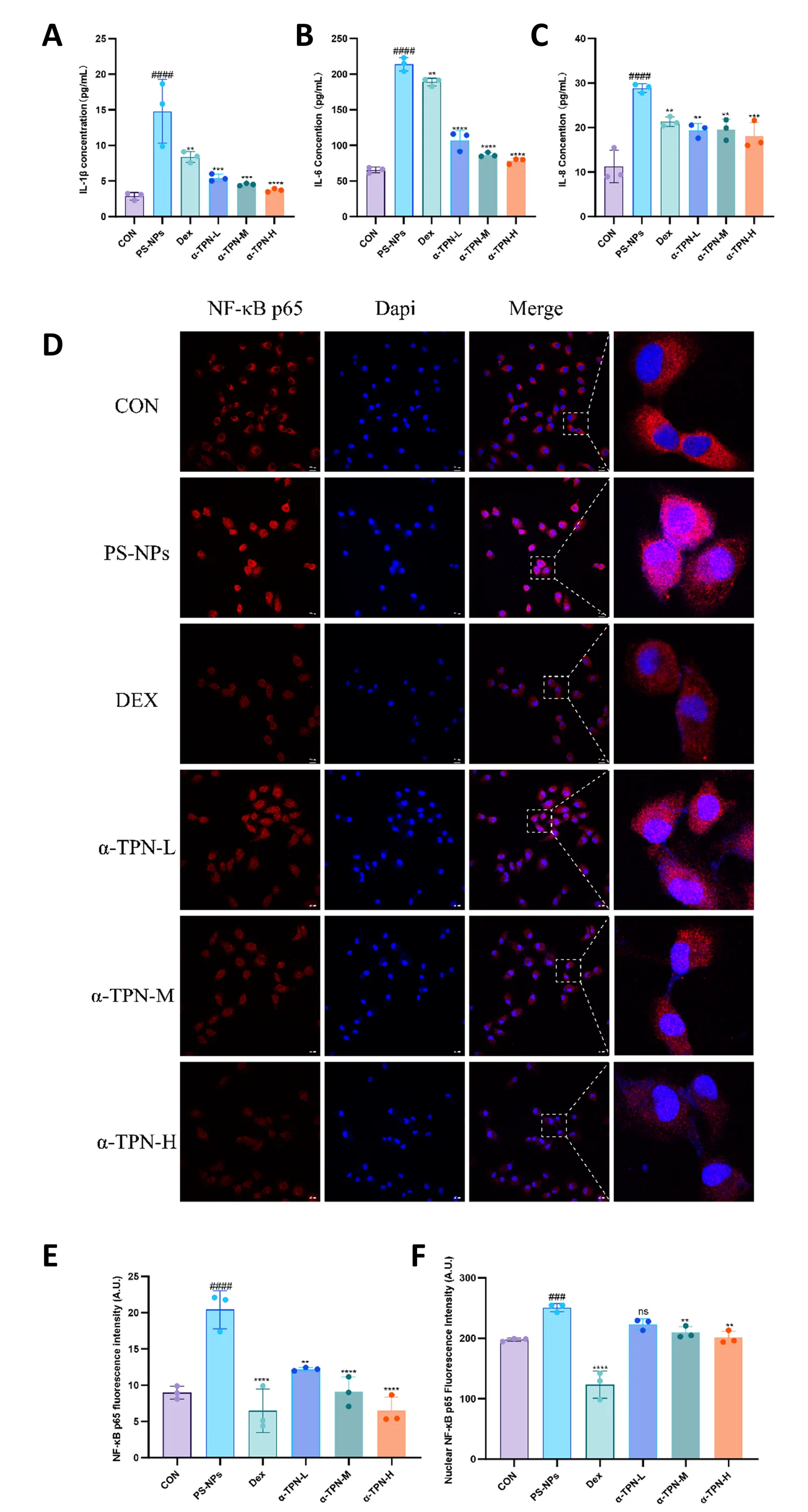
Effects of α-TPN pretreatment on PS-NP-induced inflammatory responses in A549 cells. (**A**–**C**) Levels of IL-1β, IL-6, and IL-8 in A549 cell culture supernatants measured by ELISA; (**D**) representative immunofluorescence images of NF-κB p65 in A549 cells; (**E**) quantification of overall cellular NF-κB p65 fluorescence intensity; (**F**) quantification of nuclear NF-κB p65 fluorescence intensity. ### *p* < 0.001, and #### *p* < 0.0001 indicate statistically significant differences compared with the Control group; ** *p* < 0.01, *** *p* < 0.001, and **** *p* < 0.0001 indicate statistically significant differences compared with the PS-NPs group. ns, not significant (*p* ≥ 0.05).

PS-NP exposure markedly elevated IL-1β, IL-6, and IL-8 levels in A549 cell supernatants compared with the Control group (all *p* < 0.0001). DEX treatment reduced the levels of all three cytokines (*p* < 0.01). α-TPN pretreatment also attenuated these increases. IL-1β levels decreased significantly at all three α-TPN doses (*p* < 0.001 for the low and medium doses; *p* < 0.0001 for the high dose). A similar reduction was observed for IL-6 (all *p* < 0.0001). IL-8 levels were also lower in the α-TPN-L, α-TPN-M, and α-TPN-H groups (*p* < 0.01, *p* < 0.01, and *p* < 0.001, respectively). The reductions in IL-1β and IL-6 were generally greater at higher α-TPN doses (Figure 2A–C).

NF-κB p65 expression was further examined by immunofluorescence. Compared with the Control group, PS-NP exposure increased NF-κB p65 fluorescence intensity, with significant elevations in both overall cellular and nuclear signals (*p* < 0.0001 and *p* < 0.001, respectively). DEX treatment reduced both signals compared with the PS-NP group (both *p* < 0.0001). α-TPN pretreatment also decreased overall cellular fluorescence intensity in all three dose groups (*p* < 0.01, *p* < 0.0001, and *p* < 0.0001, respectively). However, for nuclear fluorescence intensity, significant reductions were observed only in the α-TPN-M and α-TPN-H groups (both *p* < 0.01), with no significant change in the low-dose group (Figure 2D–F).

### 2.3. α-Terpineol Attenuates PS-NP-Induced NF-κB/GSDMD-Related Pyroptosis

**Figure 3:**
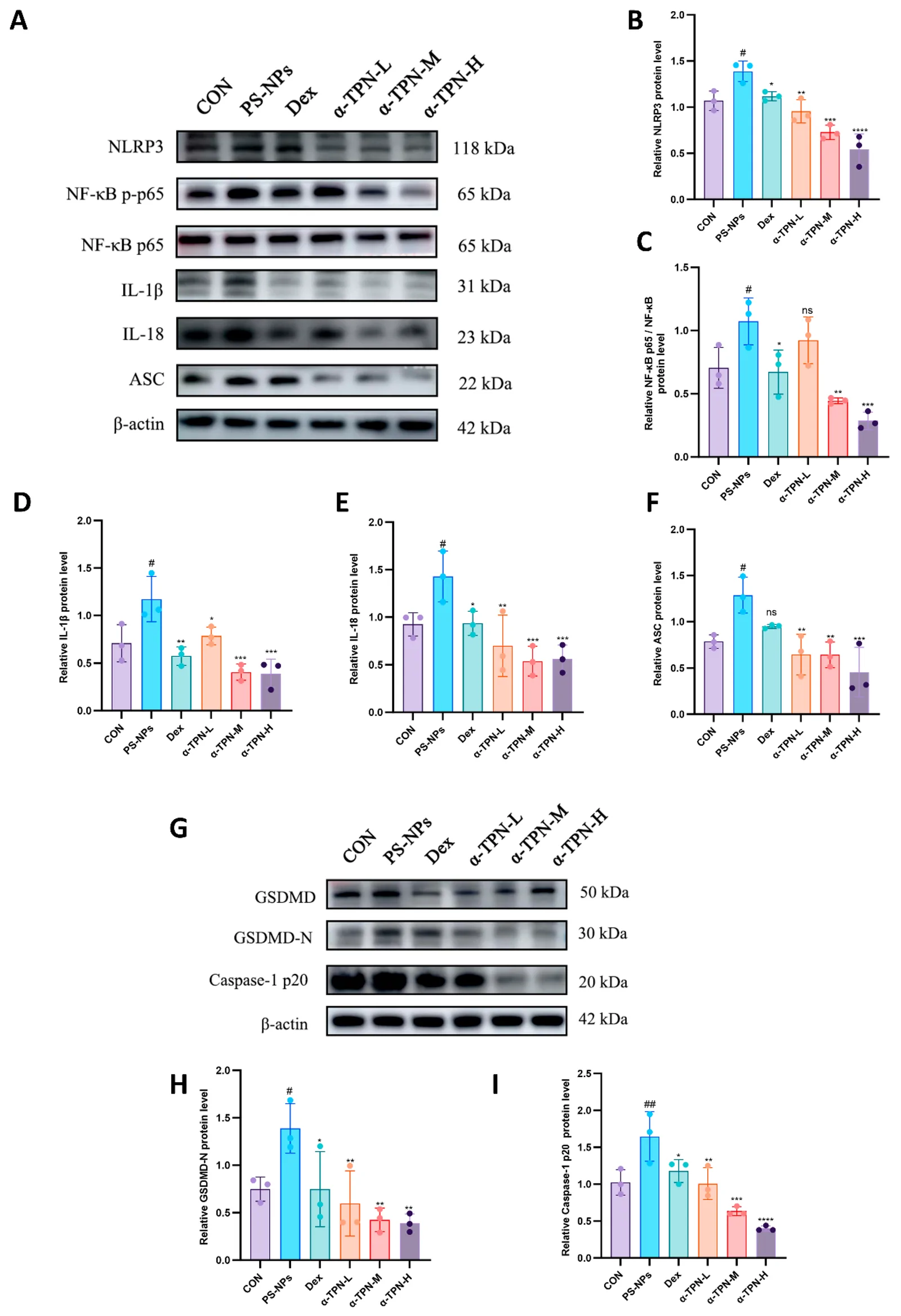
Effects of α-TPN pretreatment on PS-NP-induced NF-κB/GSDMD-related inflammatory and pyroptotic signaling in A549 cells. (**A**) Representative Western blot images of NLRP3, phospho-NF-κB p65, NF-κB p65, IL-1β, IL-18, ASC, and β-actin; (**B**–**F**) quantitative analysis of NLRP3, the p-p65/p65 ratio, IL-1β, IL-18, and ASC protein levels, respectively; (**G**) representative Western blot images of GSDMD, GSDMD-N, cleaved caspase-1 (p20), and β-actin; (**H**,**I**) quantitative analysis of GSDMD-N and cleaved caspase-1 (p20) protein levels, respectively. # *p* < 0.05, ## *p* < 0.01 indicate statistically significant differences compared with the Control group; * *p* < 0.05, ** *p* < 0.01, *** *p* < 0.001, and **** *p* < 0.0001 indicate statistically significant differences compared with the PS-NPs group. ns, not significant (*p* ≥ 0.05).

Western blotting was performed to examine NF-κB signaling and pyroptosis-related proteins in A549 cells. As shown in Figure 3A–C, PS-NP exposure increased NLRP3 expression and p65 phosphorylation compared with the Control group (both *p* < 0.05). DEX reduced NLRP3 expression and the p-p65/p65 ratio (*p* < 0.05 for both). α-TPN also decreased NLRP3 expression in all three dose groups (*p* < 0.01, *p* < 0.001, and *p* < 0.0001, respectively). The p-p65/p65 ratio was reduced by α-TPN-M and α-TPN-H (*p* < 0.01 and *p* < 0.001, respectively), but not by α-TPN-L.

PS-NP exposure resulted in higher levels of IL-1β, IL-18, and ASC than those in the Control group (all *p* < 0.05) (Figure 3D–F). DEX treatment lowered IL-1β and IL-18 levels (*p* < 0.01 and *p* < 0.05, respectively), but had no significant effect on ASC. α-TPN treatment also reduced the levels of these proteins. IL-1β decreased at all three doses (*p* < 0.05, *p* < 0.001, and *p* < 0.001, respectively), as did IL-18 (*p* < 0.01, *p* < 0.001, and *p* < 0.001, respectively). ASC levels were also lower in the α-TPN-L, α-TPN-M, and α-TPN-H groups (*p* < 0.01, *p* < 0.01, and *p* < 0.001, respectively).

Regarding pyroptosis-related proteins, PS-NP exposure increased GSDMD-N and cleaved caspase-1 (p20) levels compared with the Control group (*p* < 0.05 and *p* < 0.01, respectively) (Figure 3G–I). Both proteins were reduced by DEX (*p* < 0.05 for both). α-TPN decreased GSDMD-N levels in all three dose groups (all *p* < 0.01), while cleaved caspase-1 (p20) was reduced in the α-TPN-L, α-TPN-M, and α-TPN-H groups (*p* < 0.01, *p* < 0.001, and *p* < 0.0001, respectively).

### 2.4. α-Terpineol Improves Pulmonary Function in PS-NP-Exposed Mice

**Figure 4:**
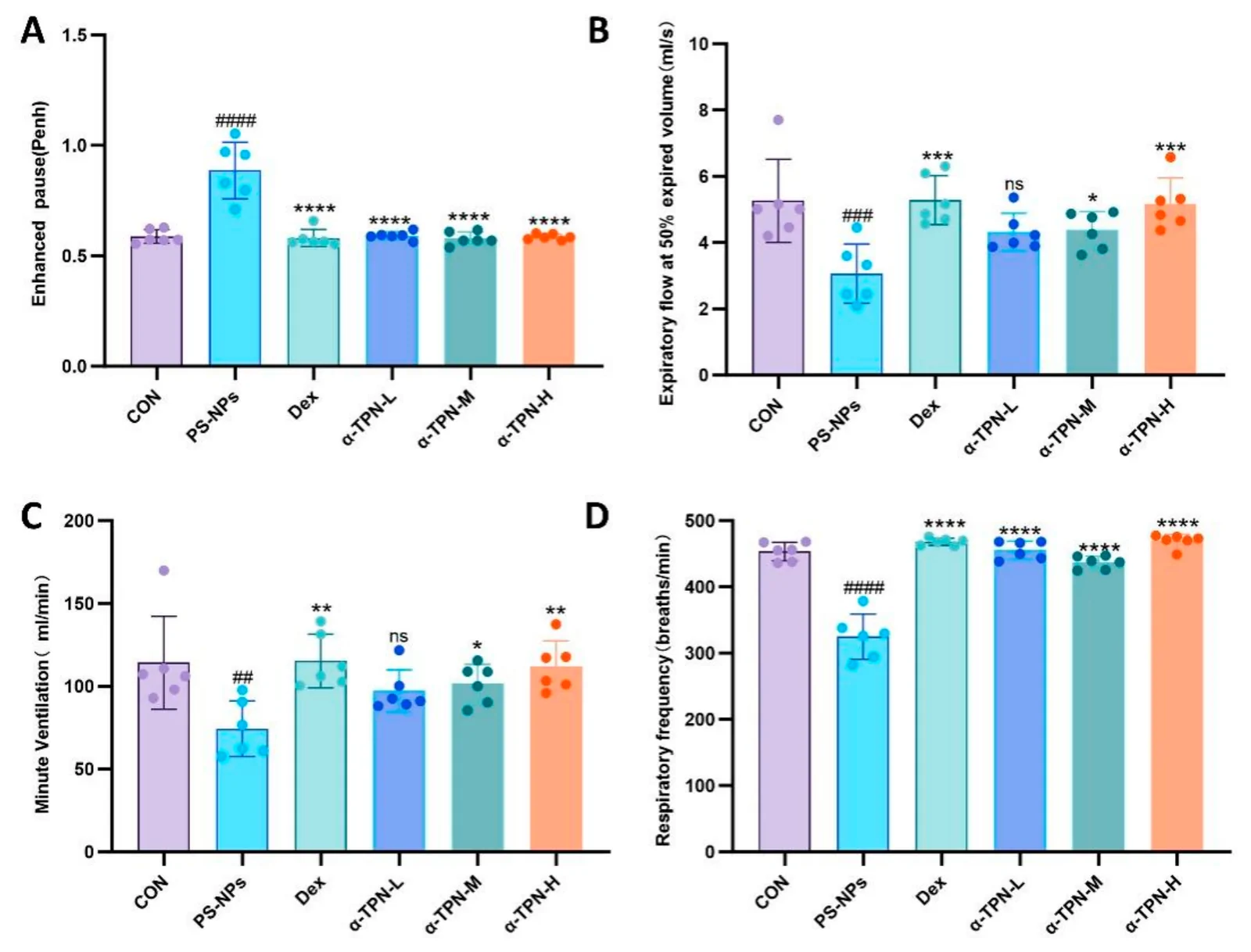
Effects of α-TPN on pulmonary function in PS-NP-exposed mice. (**A**) Enhanced pause (Penh); (**B**) expiratory flow at 50% expired volume (EF50); (**C**) minute ventilation (MV); (**D**) respiratory frequency (fbpm). ## *p* < 0.01, ### *p* < 0.001, and #### *p* < 0.0001 indicate statistically significant differences compared with the Control group; * *p* < 0.05, ** *p* < 0.01, *** *p* < 0.001, and **** *p* < 0.0001 indicate statistically significant differences compared with the PS-NPs group. ns, not significant (*p* ≥ 0.05).

Compared with the Control group, PS-NP exposure resulted in impaired pulmonary function, with a marked increase in Penh and reductions in EF50, MV, and fbpm (*p* < 0.0001, *p* < 0.001, *p* < 0.01, and *p* < 0.0001, respectively) (Figure 4A–D). DEX treatment reversed these changes, lowering Penh while increasing EF50, MV, and fbpm, with significant differences observed for all four parameters. α-TPN treatment also alleviated the pulmonary function changes caused by PS-NPs. Penh was reduced at all three doses (all *p* < 0.0001), whereas EF50 and MV increased significantly only in the medium- and high-dose groups (EF50: *p* < 0.05 and *p* < 0.001; MV: *p* < 0.05 and *p* < 0.01, respectively). fbpm was higher in all three α-TPN-treated groups than in the PS-NP group (all *p* < 0.0001) (Figure 4A–D).

### 2.5. α-Terpineol Ameliorates PS-NP-Induced Pulmonary Histopathological and Inflammatory Changes

**Figure 5:**
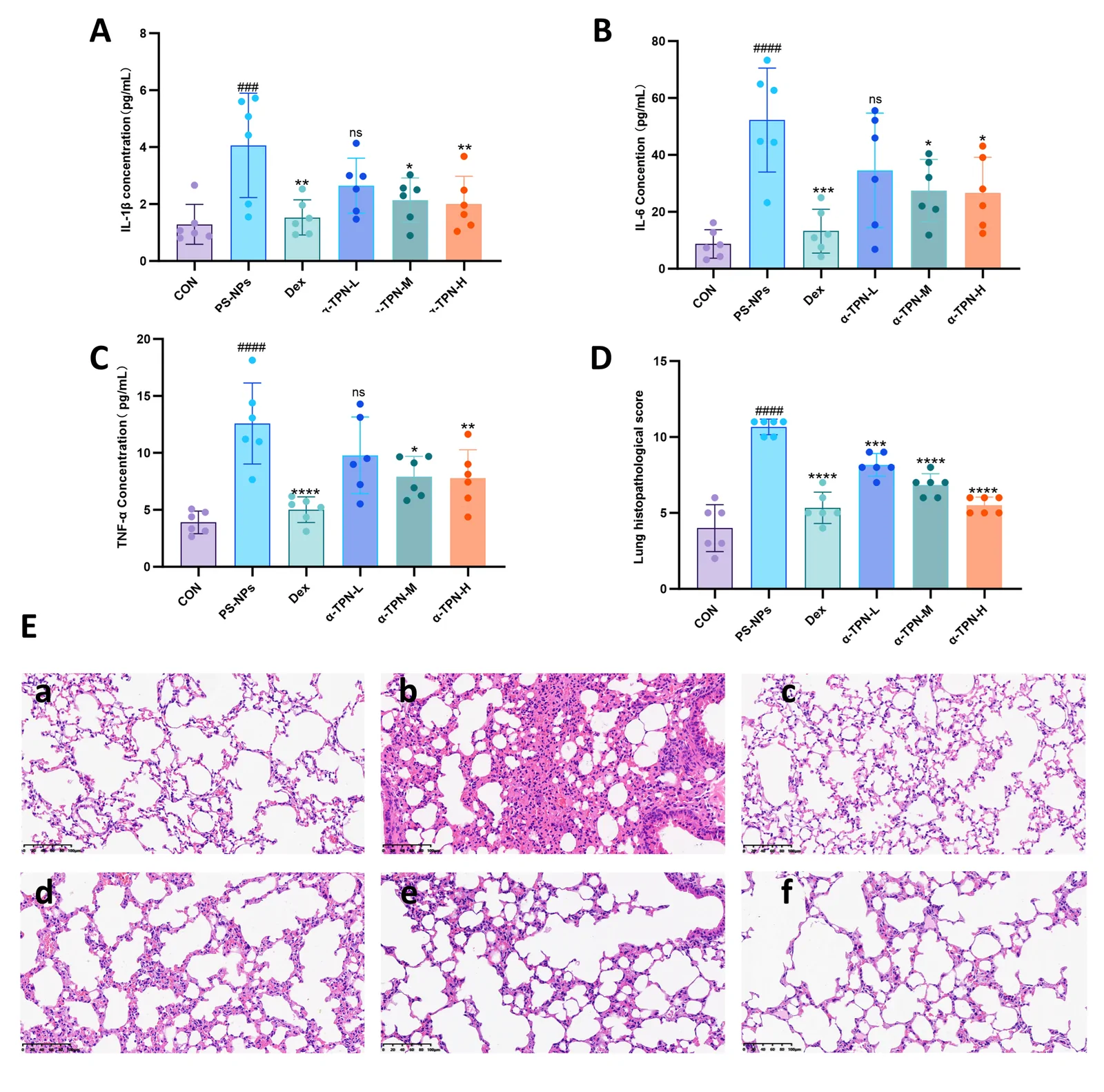
Effects of α-TPN on PS-NP-induced pulmonary histopathological and inflammatory changes in mice. (**A**–**C**) Levels of IL-1β, IL-6, and TNF-α in BALF measured by ELISA; (**D**) lung histopathological scores; (**E**) representative H&E-stained lung sections from the Control (**a**), PS-NP (**b**), DEX (**c**), α-TPN-L (**d**), α-TPN-M (**e**), and α-TPN-H (**f**) groups. Images were acquired at 20× magnification. Scale bars, 100 μm. ### *p* < 0.001, and #### *p* < 0.0001 indicate statistically significant differences compared with the Control group; * *p* < 0.05, ** *p* < 0.01, *** *p* < 0.001, and **** *p* < 0.0001 indicate statistically significant differences compared with the PS-NPs group. ns, not significant (*p* ≥ 0.05).

ELISA analysis of BALF showed higher levels of IL-1β, IL-6, and TNF-α in the PS-NP group than in the Control group (*p* < 0.001, *p* < 0.0001, and *p* < 0.0001, respectively) (Figure 5A–C). DEX treatment significantly lowered the levels of all three cytokines (*p* < 0.01, *p* < 0.001, and *p* < 0.0001, respectively). α-TPN produced similar changes, with more evident effects at the medium and high doses. Compared with the PS-NP group, α-TPN-M and α-TPN-H significantly reduced IL-1β (*p* < 0.05 and *p* < 0.01, respectively), IL-6 (both *p* < 0.05), and TNF-α (*p* < 0.05 and *p* < 0.01, respectively). No significant changes in these cytokines were detected in the α-TPN-L group.

The histological findings were consistent with the changes in inflammatory cytokines. In the PS-NP group, H&E staining showed alveolar wall thickening, inflammatory cell infiltration, alveolar edema, and disrupted alveolar structure (Figure 5E(b)). These changes were less evident following DEX treatment and α-TPN administration at medium and high doses (Figure 5E(c,e,f)). The PS-NP group also showed a significantly higher lung injury score than the Control group (*p* < 0.0001). Treatment with DEX and α-TPN significantly lowered the score compared with the PS-NP group (*p* < 0.0001 for DEX; *p* < 0.001, *p* < 0.0001, and *p* < 0.0001 for α-TPN-L, α-TPN-M, and α-TPN-H, respectively) (Figure 5D).

### 2.6. α-Terpineol Attenuates NF-κB/GSDMD-Related Changes in the Lungs of PS-NP-Exposed Mice

**Figure 6:**
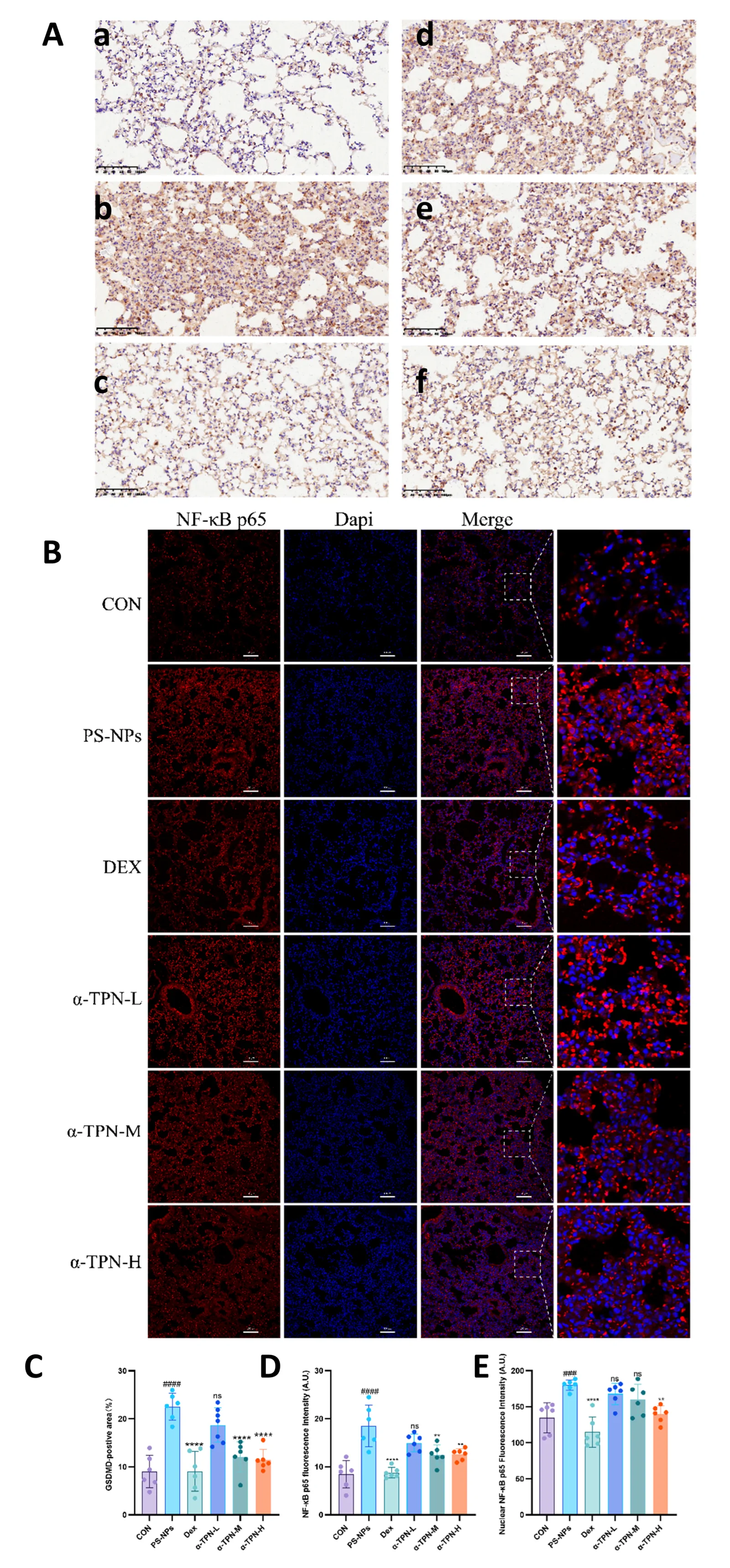
Effects of α-TPN on NF-κB/GSDMD-related changes in the lungs of PS-NP-exposed mice. (**A**) Representative immunohistochemical images of GSDMD expression in lung tissues from the Control (**a**), PS-NP (**b**), DEX (**c**), α-TPN-L (**d**), α-TPN-M (**e**), and α-TPN-H (**f**) groups; (**B**) representative immunofluorescence images of NF-κB p65; (**C**) quantitative analysis of GSDMD-positive area; (**D**) quantitative analysis of nuclear NF-κB p65 fluorescence intensity; (**E**) quantitative analysis of overall NF-κB p65 fluorescence intensity. ### *p* < 0.001, and #### *p* < 0.0001 indicate statistically significant differences compared with the Control group; ** *p* < 0.01, and **** *p* < 0.0001 indicate statistically significant differences compared with the PS-NPs group. ns, not significant (*p* ≥ 0.05).

GSDMD expression and NF-κB p65 localization in lung tissues were examined by immunohistochemistry and immunofluorescence, respectively. Immunohistochemical staining revealed a marked increase in GSDMD-positive staining following PS-NP exposure compared with the Control group (*p* < 0.0001) (Figure 6A(b),C). This increase was attenuated by DEX treatment (*p* < 0.0001)(Figure 6A(c),C) and by α-TPN at medium and high doses (both *p* < 0.0001) (Figure 6A(e,f),C), whereas the reduction in the α-TPN-L group was not statistically significant (Figure 6A(d),C).

Immunofluorescence analysis showed that PS-NP exposure significantly increased overall and nuclear NF-κB p65 fluorescence intensities compared with the Control group (*p* < 0.0001 and *p* < 0.001, respectively). DEX treatment significantly reduced both measurements (both *p* < 0.0001). Similarly, α-TPN treatment reduced overall fluorescence intensity in the α-TPN-M and α-TPN-H groups (both *p* < 0.01), whereas no significant reduction was observed in the α-TPN-L group. Regarding nuclear fluorescence intensity, a significant decrease was observed only in the α-TPN-H group (*p* < 0.01), with no significant changes in the α-TPN-L or α-TPN-M groups (Figure 6B,D,E).

### 2.7. α-Terpineol Modulates NF-κB/GSDMD-Related Pyroptosis in the Lungs of PS-NP-Exposed Mice

**Figure 7:**
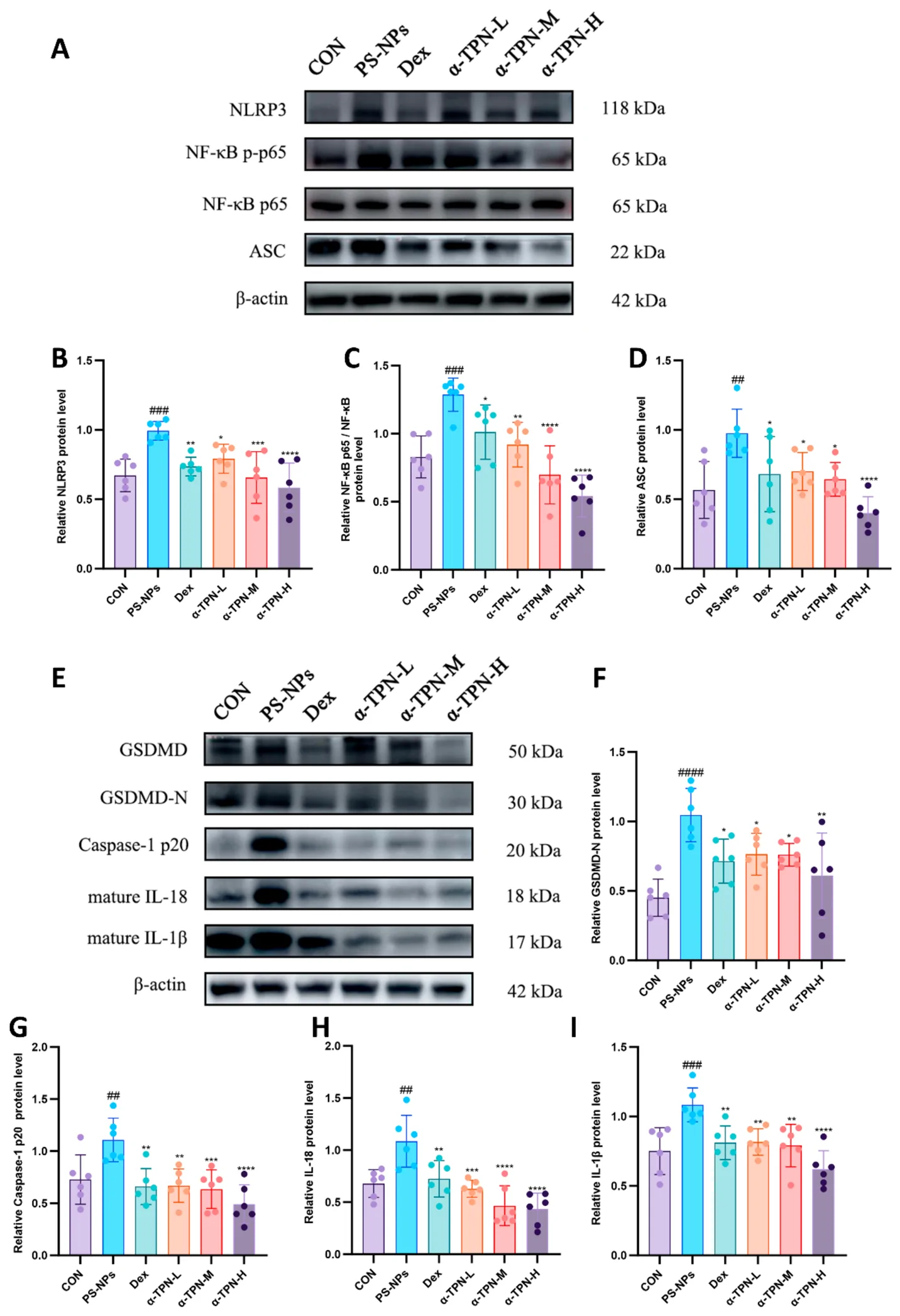
Effects of α-TPN on NF-κB- and pyroptosis-related proteins in the lungs of PS-NP-exposed mice. (**A**) Representative Western blot images of NLRP3, phospho-NF-κB p65 (p-p65), NF-κB p65, ASC, and β-actin; (**B**–**D**) quantitative analysis of NLRP3, the p-p65/p65 ratio, and ASC protein levels, respectively; (**E**) representative Western blot images of GSDMD, GSDMD-N, cleaved caspase-1 (p20), mature IL-18, mature IL-1β, and β-actin; (**F**–**I**) quantitative analysis of GSDMD-N, cleaved caspase-1 (p20), mature IL-18, and mature IL-1β protein levels, respectively. β-Actin was used as the loading control. ## *p* < 0.01, ### *p* < 0.001, and #### *p* < 0.0001 indicate statistically significant differences compared with the Control group; * *p* < 0.05, ** *p* < 0.01, *** *p* < 0.001, and **** *p* < 0.0001 indicate statistically significant differences compared with the PS-NPs group.

Western blotting was used to assess NF-κB signaling and pyroptosis-related proteins in lung tissues. Compared with the Control group, PS-NP exposure increased the p-p65/p65 ratio, NLRP3, and ASC levels (*p* < 0.001, *p* < 0.001, and *p* < 0.01, respectively) (Figure 7A–D). DEX treatment reduced NLRP3 levels, the p-p65/p65 ratio, and ASC levels compared with the PS-NP group (*p* < 0.01, *p* < 0.05, and *p* < 0.05, respectively). α-TPN also reduced p-p65/p65 ratio at all three doses (*p* < 0.01, *p* < 0.0001, and *p* < 0.0001 for the low-, medium-, and high-dose groups, respectively). NLRP3 levels were likewise decreased in the α-TPN-L, α-TPN-M, and α-TPN-H groups (*p* < 0.05, *p* < 0.001, and *p* < 0.0001, respectively). The corresponding reductions in ASC were significant in all three groups (*p* < 0.05, *p* < 0.05, and *p* < 0.0001, respectively) (Figure 7B–D).

PS-NP exposure also increased the levels of GSDMD-N, cleaved caspase-1 (p20), mature IL-18, and mature IL-1β compared with the Control group (*p* < 0.0001, *p* < 0.01, *p* < 0.01, and *p* < 0.001, respectively) (Figure 7E–I). DEX treatment significantly reduced GSDMD-N levels (*p* < 0.05), as well as cleaved caspase-1 (p20), mature IL-18, and mature IL-1β levels (all *p* < 0.01) compared with the PS-NP group. α-TPN treatment also lowered GSDMD-N levels in all three dose groups (*p* < 0.05, *p* < 0.05, and *p* < 0.01, respectively). Cleaved caspase-1 (p20) levels decreased in the α-TPN-L, α-TPN-M, and α-TPN-H groups (*p* < 0.01, *p* < 0.001, and *p* < 0.0001, respectively). Mature IL-18 levels were reduced at all three doses (*p* < 0.001, *p* < 0.0001, and *p* < 0.0001, respectively). Mature IL-1β levels also decreased in each α-TPN group, with *p* < 0.01 for the low- and medium-dose groups and *p* < 0.0001 for the high-dose group (Figure 7F–I).

## 3. Discussion

Nanoplastics have attracted increasing attention as emerging environmental contaminants because of their small size, large specific surface area, and potential to cross biological barriers [16]. The respiratory tract represents an important route of human exposure to airborne plastic particles. Once deposited in the lungs, NPs may interact with pulmonary epithelial cells and trigger oxidative stress, inflammation, and cellular injury [17,18]. Previous studies have shown that PS-NP exposure can induce oxidative damage, pulmonary inflammation, epithelial barrier disruption, and increased production of inflammatory cytokines [19,20]. These effects may be partly associated with excessive reactive oxygen species (ROS) generation and impaired redox homeostasis, which can damage cellular components and activate inflammatory pathways [21,22]. Persistent oxidative and inflammatory responses may further compromise epithelial integrity, promote inflammatory cell infiltration, and impair lung structure and function [23]. Consistent with these findings, PS-NP exposure in the present study caused cellular injury in A549 cells and pulmonary pathological and inflammatory changes in mice, accompanied by alterations in NF-κB/GSDMD-related signaling. α-Terpineol treatment attenuated these changes, possibly by regulating inflammatory responses and GSDMD-related pyroptosis.

In this study, the basic physicochemical characteristics of the PS-NPs were provided by the manufacturer. However, we did not independently examine the PS-NPs for residual chemicals, endotoxin, or other contaminants. Therefore, a possible contribution of these impurities to the inflammatory responses observed in this study cannot be excluded. Further studies involving comprehensive purity assessment and contaminant analysis are needed to distinguish the biological effects of PS-NPs from those potentially associated with impurities.

Despite these findings, the relevance of the exposure conditions to real-world human exposure should be considered. Although microplastics have been detected in indoor and outdoor air, quantitative information on airborne nanoplastic concentrations, particularly those capable of reaching the lower respiratory tract, remains limited. This makes it difficult to directly compare experimental exposure levels with actual environmental or occupational exposures [24,25]. The PS-NP dose used in the mouse experiments was selected to establish an experimental model of pulmonary injury rather than to represent typical human inhalation exposure. Similarly, the concentrations used in the A549 cell experiments cannot be directly translated into human inhalation doses. Therefore, the findings should be interpreted in the context of the experimental exposure conditions, and further studies using environmentally relevant doses are needed to clarify the potential health effects of PS-NPs under realistic exposure scenarios.

NF-κB signaling is an important regulator of inflammatory responses and innate immunity, and its activation is closely associated with tissue injury induced by environmental stressors [26,27]. Under resting conditions, NF-κB remains predominantly in the cytoplasm through its interaction with IκB proteins. Upon stimulation, IκB phosphorylation and degradation allow NF-κB, particularly the p65 subunit, to translocate into the nucleus and regulate the expression of inflammatory mediators [28,29]. Previous studies have also linked exposure to particulate pollutants and nanoplastics with activation of NF-κB-related signaling and increased inflammatory mediator production in the respiratory system [30,31]. Following PS-NP treatment, higher levels of inflammatory cytokines were detected in A549 cell culture supernatants and mouse BALF. The alterations in p65 immunofluorescence signals and related protein levels also indicated the involvement of NF-κB signaling in the inflammatory responses elicited by PS-NPs. The resulting increase in inflammatory mediators may further aggravate local inflammation and promote lung injury.

Pyroptosis is a form of programmed cell death closely associated with inflammatory tissue injury [32]. It involves the activation of inflammatory caspases and the subsequent cleavage of gasdermin D (GSDMD). The resulting N-terminal fragment (GSDMD-N) forms pores in the plasma membrane, allowing the release of mature IL-1β and IL-18 [33]. Among the pathways involved in this process, the NLRP3 inflammasome, which comprises NLRP3, ASC, and caspase-1, is a well-established mediator of canonical pyroptosis [34,35]. Previous studies have reported that particulate matter, engineered nanoparticles, and microplastic-derived particles can trigger inflammasome-related responses and GSDMD-associated cell death [36,37]. In this study, PS-NP exposure was associated with alterations in inflammasome-related proteins and increased levels of pyroptosis-associated markers in A549 cells and mouse lung tissues. These changes, along with enhanced GSDMD immunostaining and elevated inflammatory cytokine levels, support the involvement of pyroptosis-related processes in PS-NP-induced pulmonary inflammation. Although Annexin V/PI staining cannot specifically identify pyroptosis, the accompanying changes in GSDMD-N, cleaved caspase-1, and mature inflammatory cytokines provide further evidence of pyroptosis-associated responses. Overall, GSDMD-related pyroptotic processes may be involved in the pulmonary inflammatory injury caused by PS-NPs.

NF-κB signaling and pyroptosis are interconnected processes that may jointly contribute to inflammatory tissue injury. Activation of NF-κB can promote the transcription of inflammasome components and pro-inflammatory cytokine precursors, thereby providing the necessary conditions for subsequent inflammasome activation and pyroptotic responses [38,39]. Conversely, pyroptotic cell death may further intensify inflammatory signaling through the release of intracellular danger signals and inflammatory mediators, potentially establishing a self-amplifying cycle [40,41]. In the present study, PS-NP exposure induced concurrent alterations in NF-κB signaling and pyroptosis-related markers in both A549 cells and mouse lung tissues. The changes observed across these two models suggest that NF-κB-related inflammatory responses and GSDMD-associated pyroptosis may be linked during PS-NP-induced pulmonary injury. Nevertheless, the absence of pathway-specific inhibition, gene silencing, or rescue experiments limits the ability to establish a direct regulatory relationship between NF-κB signaling and GSDMD-associated pyroptosis. Thus, the involvement of the NF-κB/GSDMD signaling axis in PS-NP-induced lung injury remains a potential mechanism that requires further investigation.

Natural compounds have gained increasing interest for their ability to modulate oxidative stress and inflammation [42]. α-Terpineol is a naturally occurring monoterpene alcohol with reported anti-inflammatory, antioxidant, and cytoprotective effects in various experimental models [43,44,45]. However, the protective effects of α-TPN against nanoplastic-induced lung injury have not been adequately investigated. In the present study, α-TPN treatment alleviated PS-NP-induced cellular injury and inflammatory responses in A549 cells, accompanied by changes in NF-κB- and pyroptosis-related markers. The extent of these responses varied across treatment concentrations and experimental endpoints, with more consistent effects generally observed at medium and high doses. These findings indicate that α-TPN can attenuate PS-NP-induced cellular injury, although its effects may differ depending on the biological process examined.

The present findings suggest that NF-κB-related signaling and GSDMD-associated pyroptosis may be involved in the protective effects of α-TPN against PS-NP-induced pulmonary injury. However, the current evidence does not establish these pathways as direct mediators of α-TPN action. Although changes in NF-κB-related proteins and pyroptosis-associated markers were observed following α-TPN treatment, no pathway-specific inhibition, gene silencing, or rescue experiments were performed to verify their causal involvement. Therefore, these findings support an association between the modulation of these processes and the protective effects of α-TPN, rather than demonstrating that inhibition of NF-κB signaling or GSDMD-mediated pyroptosis is directly responsible for the observed benefits.

Notably, the expression of most of the examined inflammation- and pyroptosis-related proteins in the medium- and high-dose α-TPN groups was lower than that in the Control group. This pattern indicates that the changes following combined PS-NP exposure and α-TPN treatment were not limited to restoring the elevated protein levels toward those observed under normal conditions. Instead, the expression of several proteins showed a more pronounced reduction. Similar decreases in multiple pyroptosis-associated proteins following pharmacological intervention have been reported in experimental models [46,47]. Nevertheless, since an α-TPN-only group was not included in the present study, it remains difficult to determine whether the lower protein levels reflect an effect of α-TPN on basal expression or other responses associated with the combined treatment. Therefore, these findings should be interpreted cautiously, particularly with regard to their biological significance.

GSDMD immunohistochemistry was performed to examine its distribution in lung tissues, while other inflammasome- and pyroptosis-related proteins were assessed by Western blotting. Immunofluorescence staining of total p65 was used to evaluate its nuclear localization, whereas Western blotting was performed to determine total and phosphorylated p65 levels in lung tissue lysates. Although nuclear translocation of total p65 is commonly associated with NF-κB activation, different phosphorylation forms of p65 may exhibit distinct subcellular distributions. For example, p65 phosphorylated at Ser468 has been reported to accumulate predominantly in the nucleus, whereas Ser536-phosphorylated p65 is mainly localized in the cytoplasm [48]. Therefore, nuclear accumulation of total p65 does not necessarily reflect a specific phosphorylation state. This may explain why increased nuclear p65 fluorescence was observed following PS-NP exposure despite no significant change in total p65 abundance detected by Western blotting. The changes in p-p65 levels provide additional information regarding p65 phosphorylation. Nevertheless, since phosphorylated p65 was not examined by immunofluorescence, its subcellular distribution and relationship with total p65 nuclear localization could not be directly determined.

Dexamethasone’s known anti-inflammatory properties led to its usage as a positive control. DEX enhanced lung function and decreased inflammatory cytokine levels and histopathological alterations in mice exposed to PS-NPs, along with alterations in a number of proteins linked to pyroptosis and NF-κB. There were variations in the degree of change and statistical significance among the various markers, indicating that the molecular responses were not consistent. Following α-terpineol administration, a similar trend was seen, especially across the various dosages. This might be because diverse features of the reaction to exposure to PS-NPs are reflected in functional, inflammatory, histological, and molecular measures. Although the effects varied by endpoint, α-terpineol caused alterations that were comparable to those seen with DEX across a number of functional and inflammatory markers. These results provide more evidence that α-terpineol may be able to reduce lung damage caused by PS-NPs.

Several limitations of this study should be considered. First, although alterations in NF-κB-related signaling and GSDMD-associated pyroptosis were observed following PS-NP exposure and α-terpineol treatment, the current findings do not establish whether these pathways are directly responsible for the protective effects of α-terpineol. Further mechanistic studies using pathway inhibitors, GSDMD knockdown, and rescue approaches are needed to clarify their involvement. Second, A549 cells, which are derived from human lung adenocarcinoma, may not fully reproduce the biological characteristics or responses of normal alveolar epithelial cells, despite their widespread application in pulmonary research [49]. Confirmation using primary human alveolar epithelial cells or other non-tumorigenic lung models would help establish the relevance of these in vitro findings. Third, the deposition and distribution of PS-NPs in lung tissues and cells were not directly examined. Particle-tracking studies may provide further insight into the relationship between particle localization and the resulting pulmonary effects. In the present study, only male mice were included to maintain consistency within the animal experimental model. However, sex-related differences may influence responses to environmental particles and inflammatory stimuli. Therefore, the present findings cannot establish whether female mice respond differently to PS-NP exposure or α-terpineol treatment. Future studies involving both sexes are needed to investigate potential sex-related differences in pulmonary injury and treatment responses. In addition, further pharmacokinetic and pharmacological studies are required to better characterize α-terpineol and assess its potential for future application.

In summary, PS-NPs caused GSDMD-related pyroptosis, inflammation, and lung damage. α-terpineol reduced histological alterations, lung dysfunction, and cellular damage, possibly via regulating inflammatory and pyroptotic reactions linked to NF-κB/GSDMD. These results demonstrate the potential of α-terpineol as a natural substance for reducing lung damage caused by PS-NPs.

## 4. Materials and Methods

### 4.1. Materials

#### 4.1.1. Main Reagents and Materials

**Figure 8:**
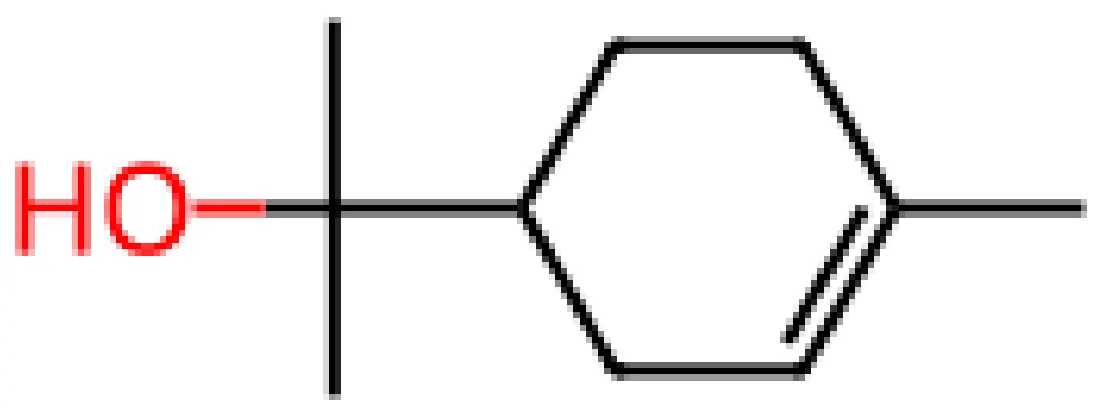
Chemical structure of α-terpineol.

Polystyrene nanoplastics (approximately 80 nm in diameter; density, 1.05 g/cm^3^; coefficient of variation, <3%) were purchased from Zhongke Leiming (Beijing) Technology Co., Ltd. (Beijing, China). α-terpineol (purity: 98–99%; CAS No. 98-55-5) was purchased from Shanghai Macklin Biochemical Technology Co., Ltd. (Shanghai, China), the chemical structure of α-terpineol is presented in Figure 8. Dexamethasone (DEX; purity, ≥99%; Cat. No. D80001) was purchased from Psaitong (Shanghai, China). L-lactate dehydrogenase (L-LDH) assay kit (Cat. No. BC0685) was purchased from Beijing Solarbio Science & Technology Co., Ltd. (Beijing, China). Annexin V-FITC/PI Apoptosis Detection Kit (Cat. No. RK05875) was obtained from ABclonal Technology Co., Ltd. (Wuhan, China). ELISA kits for the detection of inflammatory cytokines were also obtained from ABclonal Technology Co., Ltd. (Wuhan China). Servicebio Technology Co., Ltd. (Wuhan, China) provided a 5× SDS-PAGE protein loading buffer (Cat. No. G2075) and a bicinchoninic acid (BCA) protein test kit (Cat. No. G2026). We bought the DAPI staining solution (Cat. No. C1005) from Beyotime Biotechnology in Shanghai, China.

The primary antibodies used for Western blotting, immunofluorescence, and immunohistochemistry included NLRP3 polyclonal antibody (Cat. No. 30109-1-AP), NF-κB p65 recombinant monoclonal antibody (Cat. No. 80979-1-RR), phospho-NF-κB p65 (Ser536) recombinant monoclonal antibody (Cat. No. 80379-2-RR), and ASC/TMS1 polyclonal antibody (Cat. No. 10500-1-AP), which were purchased from Wuhan Sanying Biotechnology Co., Ltd. (Proteintech, Wuhan, China). Anti-IL-1β antibody (Cat. No. AF5103), anti-cleaved caspase-1 (Asp296, p20) antibody (Cat. No. AF4005), and anti-IL-18 antibody (Cat. No. DF6252) were purchased from Affinity Biosciences (Changzhou, China). Anti-cleaved N-terminal GSDMD antibody (EPR20829-408; Cat. No. ab215203) and anti-GSDMD antibody (EPR20859; Cat. No. ab219800) were purchased from Abcam (Cambridge, UK). β-Actin Rabbit mAb (Cat. No. AC050) was purchased from ABclonal Technology Co., Ltd. (Wuhan, China). HRP-conjugated goat anti-rabbit IgG polyclonal antibody (Cat. No. HA1001) was purchased from HUABIO (Hangzhou, China). GF555-labeled goat anti-mouse IgG secondary antibody (Cat. No. LF109/LF109S) was purchased from Epizyme Biomedical Technology Co., Ltd. (Shanghai, China).

The Shanghai Cell Bank of the Chinese Academy of Sciences (Shanghai, China) provided the human lung adenocarcinoma epithelial cell line A549. Meilunbio (Dalian, China) supplied fetal bovine serum (FBS; Cat. No. PWL001), 0.25% trypsin-EDTA solution (Cat. No. MA0232), and Dulbecco’s modified Eagle’s medium (DMEM, high glucose; Cat. No. MA0212-2).

Male C57BL/6J mice of specific pathogen-free (SPF) grade were purchased from SPF (Beijing) Biotechnology Co., Ltd. (Beijing, China).

#### 4.1.2. Main Instruments and Equipment

The main instruments used in this study included an inverted fluorescence microscope (Ts2-FL; Nikon, Japan), an upright fluorescence microscope (PA53FS6 + Moticam; Motic, China), a multimode microplate reader (Synergy H1; BioTek, USA), a confocal laser scanning microscope (AX; Nikon, Japan), a flow cytometer (ID7000; Sony Biotechnology, Japan), a noninvasive whole-body pulmonary function testing system for small animals (Beijing Huironghe Technology Co., Ltd., China) and a nose-and-mouth exposure system for rodents (Shanghai Yuyan Scientific Instrument Co., Ltd., China). Western blotting was performed using an Amersham 680 imaging system (GE, USA), a PowerPac Basic power supply, a Mini Trans-Blot electrophoretic transfer system, and a Mini-PROTEAN Tetra electrophoresis system (Bio-Rad, USA).

### 4.2. Cell Experiments

#### 4.2.1. Preparation of Experimental Reagents

PS-NPs were supplied as a 50 mg/mL aqueous dispersion. Before use, the stock dispersion was thoroughly mixed and diluted with the appropriate vehicle according to the experimental requirements.

α-Terpineol (100 mg) was dissolved in 1 mL of DMSO to prepare a 100 mg/mL stock solution.

Dexamethasone (DEX, 3.92 mg) was dissolved in 1 mL of DMSO to prepare a 10 mM stock solution (3.92 mg/mL).

The PS-NP stock dispersion was diluted with culture medium to obtain concentrations ranging from 5 to 500 μg/mL. The α-TPN stock solution was diluted with culture medium to the required concentrations (6.25–800 μg/mL), while DEX was diluted to a final working concentration of 10 nM (3.92 ng/mL). The final DMSO concentration in the culture medium was maintained below 0.1% (*v*/*v*).

#### 4.2.2. Cell Counting Kit-8 Assay

A549 cells were seeded into 96-well plates at a density of 5 × 10^3^ cells/mL, with 100 μL of cell suspension added per well. After cell attachment, the cells were exposed to α-TPN (6.25, 12.5, 25, 50, 100, 200, 400, 800 μg/mL) or PS-NPs (5, 25, 50, 100, 200, 300, 400, 500 μg/mL) for 24 h. Using the CCK-8 test, cell viability was evaluated by measuring absorbance at 450 nm following a one-hour incubation period at 37 °C with the reagent. Based on these results and preliminary findings, appropriate doses for further tests were chosen. Cell viability was expressed in relation to the untreated control.

#### 4.2.3. Measurement of LDH Activity

For the LDH assay, A549 cells were collected during the logarithmic growth phase and distributed into 6-well plates at 3 × 10^4^ cells per well. Once the cells had attached, the culture medium was replaced with medium containing 1% FBS and PS-NPs at the indicated concentrations (50–500 μg/mL). Triton X-100-treated cells served as the positive control for LDH release. At the end of the exposure period, the culture supernatants were collected, and LDH activity was determined using an L-LDH assay kit following the manufacturer’s protocol. Absorbance was measured with a multimode microplate reader. The results were considered alongside preliminary experimental observations to select the PS-NP concentration for subsequent experiments.

#### 4.2.4. Cell Grouping

Six groups of A549 cells were established: Control, PS-NPs, PS-NPs + DEX, PS-NPs + α-TPN-L, PS-NPs + α-TPN-M, and PS-NPs + α-TPN-H. Based on the CCK-8 and LDH assay results, PS-NPs and α-TPN were used at concentrations of 300 μg/mL and 6.25, 12.5, and 25 μg/mL, respectively. DEX was used as a positive control at a final concentration of 10 nM (3.92 ng/mL). Cells in the DEX and α-TPN groups were pretreated with the respective compounds for 6 h, after which the medium was replaced with fresh medium containing PS-NPs for a further 24 h. The PS-NP group was exposed to PS-NPs for 24 h without pretreatment, while the Control group was maintained in complete medium throughout the experiment, with medium replacement at the corresponding time point.

#### 4.2.5. Annexin V-FITC/PI Flow Cytometry

A549 cells were treated in line with the aforementioned experimental grouping after being seeded into 6-well plates. Following treatment, adherent and floating cells were gathered, cleaned with PBS, and then resuspended in binding buffer. As directed by the manufacturer, the cells were stained with PI and Annexin V-FITC using an Annexin V-FITC/PI Apoptosis Detection Kit. After the tagged cells were cultured in the dark, they were examined using a flow cytometer. Cell death was measured using the percentages of Annexin V/PI-positive cells.

#### 4.2.6. Measurement of Inflammatory Cytokines

The levels of IL-1β, IL-6, and IL-8 in the supernatants of A549 cell cultures were measured using ELISA kits in accordance with the manufacturer’s instructions.

### 4.3. Animal Experiments

All animal experiments were approved by the Animal Ethics Committee of Kunming Medical University (Approval No. kmmu20240714, approved on 1 February 2024) and were conducted in accordance with the relevant institutional guidelines for the care and use of laboratory animals.

#### 4.3.1. Animals and Experimental Grouping

Male SPF C57BL/6J mice were employed. The animals were kept in standardized housing with a 12-h light/dark cycle, controlled humidity and temperature, and unlimited access to food and drink. Following acclimatization, 36 mice were divided into six experimental groups (*n* = 6) at random: control, PS-NPs, PS-NPs + DEX, and PS-NPs with low-, medium-, or high-dose α-terpineol (α-TPN-L, α-TPN-M, and α-TPN-H, respectively).

#### 4.3.2. PS-NP Exposure and Drug Administration

The animal study design was informed by previous studies on the pulmonary toxicity of polystyrene microplastics and nanoplastics, as well as our preliminary experimental findings. In our earlier work, intratracheal instillation of 1-μm polystyrene microplastics (PS-MPs) induced lung injury in mice, while other studies have reported pulmonary toxicity following respiratory exposure to PS-NPs [50]. Based on these findings, a PS-NP dose of 15 mg/kg was selected for the present study. Considering the volatile nature of α-terpineol, nebulization was selected as the administration route in the present study. This approach was supported by previous studies demonstrating the feasibility of aerosol-based drug delivery in animal models [51]. In addition, a previously described device for low-dose intrapulmonary drug delivery in mice employed a similar aerosol-generation and delivery principle to that used in our study [52], further supporting the feasibility of this administration approach.

The experiment was carried out in two stages. From days 1 to 14, mice in the PS-NPs, PS-NPs + DEX, and PS-NPs + α-TPN groups received PS-NPs at 15 mg/kg by nose-and-mouth nebulization once daily. Control mice received the same volume of normal saline. From days 15 to 28, the control and PS-NP groups received normal saline, while the positive-control group received DEX at 1 mg/kg. The three α-TPN groups received doses of 8, 16, and 32 mg/kg, corresponding to the low-, medium-, and high-dose groups, respectively. All treatments were delivered by nebulization. The α-TPN dose range was chosen with reference to the results of the cell experiments. This second stage was intended to assess the effects of α-TPN after the PS-NP exposure period.

#### 4.3.3. Pulmonary Function Tests

A noninvasive whole-body plethysmography device for small animals was used to assess pulmonary function. Mice were given time to get used to the chamber before being measured individually while they were at rest. Minute ventilation (MV), enhanced pause (Penh), expiratory flow at 50% of the expired volume (EF50), and breathing frequency (fbpm) were among the respiratory characteristics that were measured. All measurements were carried out in compliance with the manufacturer’s instructions, and only stable recordings were kept for further examination.

#### 4.3.4. Animal Euthanasia and Sample Collection

At the end of the experiment, the mice were anesthetized and sacrificed in accordance with the approved protocol. After that, the lungs were carefully removed. Lung tissues were obtained for further histological, immunohistochemical, immunofluorescence, and Western blot analyses, and bronchoalveolar lavage fluid (BALF) was collected for the detection of inflammatory cytokines.

#### 4.3.5. BALF Collection and Inflammatory Cytokine Measurement

Following euthanasia, the left lung was ligated and collected for H&E staining, immunohistochemistry, immunofluorescence, and Western blot analyses. The right lung was lavaged with sterile PBS through a cannula inserted into the trachea to collect BALF. The recovered BALF was centrifuged at 1500× *g* for 10 min at 4 °C, and the supernatant was collected and stored at −80 °C until further analysis. The levels of TNF-α, IL-1β, and IL-6 in BALF were measured using ELISA kits according to the manufacturers’ instructions.

#### 4.3.6. Histopathological Examination

Lung tissues were fixed in 4% paraformaldehyde, embedded in paraffin, and sectioned at 4 μm. Histological changes were examined under a light microscope following H&E staining. Histopathological assessment was performed based on previously published methods for experimental lung injury [53,54]. Lung injury was evaluated using a semiquantitative scoring system comprising four parameters: alveolar septal thickening, inflammatory cell infiltration, alveolar edema, and structural damage. Each parameter was scored from 0 to 3 according to the severity of the observed lesions, with 0 indicating no apparent abnormality and scores of 1, 2, and 3 indicating mild, moderate, and severe injury, respectively. The scoring criteria are presented in Table 1. The four individual scores were summed to obtain a total lung injury score ranging from 0 to 12, with higher scores indicating more severe injury. All lung sections were independently assessed by a single investigator who was blinded to the experimental group allocation and followed the predefined scoring criteria.

#### 4.3.7. Immunohistochemistry

Lung sections encased in paraffin underwent deparaffinization, rehydration, and antigen retrieval. The slices were treated with 3% hydrogen peroxide and then incubated overnight at 4 °C with anti-GSDMD antibody (1:1000). After that, the slides were incubated with a secondary antibody, developed using DAB, and counterstained with hematoxylin. After mounting and dehydration, light microscopy was used to capture pictures, and ImageJ (version 1.54u8) was used to quantify GSDMD-positive staining.

### 4.4. Immunofluorescence Staining

Immunofluorescence staining was performed in both A549 cells and mouse lung tissues. For A549 cells, cells were cultured on glass coverslips and subjected to the designated treatments. After washing with PBS, the cells were fixed in 4% paraformaldehyde for 15 min and permeabilized with 0.1% Triton X-100 for 10 min at room temperature. Nonspecific binding was blocked with 5% bovine serum albumin for 1 h. The cells were then incubated overnight at 4 °C with an anti-NF-κB p65 primary antibody (1:250), followed by incubation with a fluorescently labeled secondary antibody for 1 h at room temperature in the dark. After DAPI counterstaining, fluorescence images were captured using a fluorescence microscope.

For mouse lung tissues, paraffin-embedded sections were deparaffinized, rehydrated, subjected to antigen retrieval, permeabilized, and blocked. The sections were incubated overnight at 4 °C with an anti-NF-κB p65 primary antibody (1:250), followed by incubation with a fluorescently labeled secondary antibody for 1 h at room temperature in the dark. Nuclei were counterstained with DAPI, and fluorescence images were acquired using microscopy. ImageJ software was used to quantify the fluorescence signals.

### 4.5. Western Blot Analysis

Western blot analysis was performed using protein extracts from A549 cells and mouse lung tissues. For A549 cells, proteins were extracted on ice using lysis buffer containing protease and phosphatase inhibitors. After sonication and centrifugation at 12,000 rpm for 15 min at 4 °C, protein concentrations were determined using a BCA assay. For mouse lung tissues, total proteins were extracted following the same procedure.

Equal amounts of protein were mixed with 5× loading buffer, heated at 100 °C for 10 min, separated by SDS-PAGE, and transferred onto PVDF membranes. After blocking, the membranes were incubated overnight at 4 °C with primary antibodies against NLRP3 (1:2000), ASC (1:5000), NF-κB p65 (1:5000), phospho-NF-κB p65 (1:2000), GSDMD (1:1000), GSDMD-N (1:1000), cleaved caspase-1 (p20) (1:2000), IL-1β (1:2000), and IL-18 (1:2000) for A549 cell samples. For mouse lung tissue samples, the primary antibodies included NLRP3 (1:2000), ASC (1:5000), NF-κB p65 (1:5000), phospho-NF-κB p65 (1:2000), GSDMD (1:1000), cleaved caspase-1 (p20) (1:2000), IL-1β (1:2000), and IL-18 (1:2000).

After washing, the membranes were incubated with HRP-conjugated secondary antibodies for 2 h at room temperature. The secondary antibody dilution was 1:50,000 for A549 cell samples and 1:5000 for mouse lung tissue samples. Protein bands were detected using a multifunctional imaging system, with β-actin serving as the loading control. Band intensities were quantified using ImageJ software.

### 4.6. Statistical Analysis

At least three independent runs of each experiment were conducted. The information is displayed as mean ± standard deviation (SD). ImageJ software (Version 1.54p, National Institutes of Health, Bethesda, MD, USA) was used to quantify Western blot band intensities, immunohistochemistry staining, and immunofluorescence signals. GraphPad Prism 9.0 was used for data visualization, and Adobe Illustrator was used to create the figures. SPSS 21.0 was used to do statistical analyses. One-way analysis of variance (ANOVA) was used to examine differences between several groups. Statistical significance was defined as a *p* value of less than 0.05.

## 5. Conclusions

**Figure 9:**
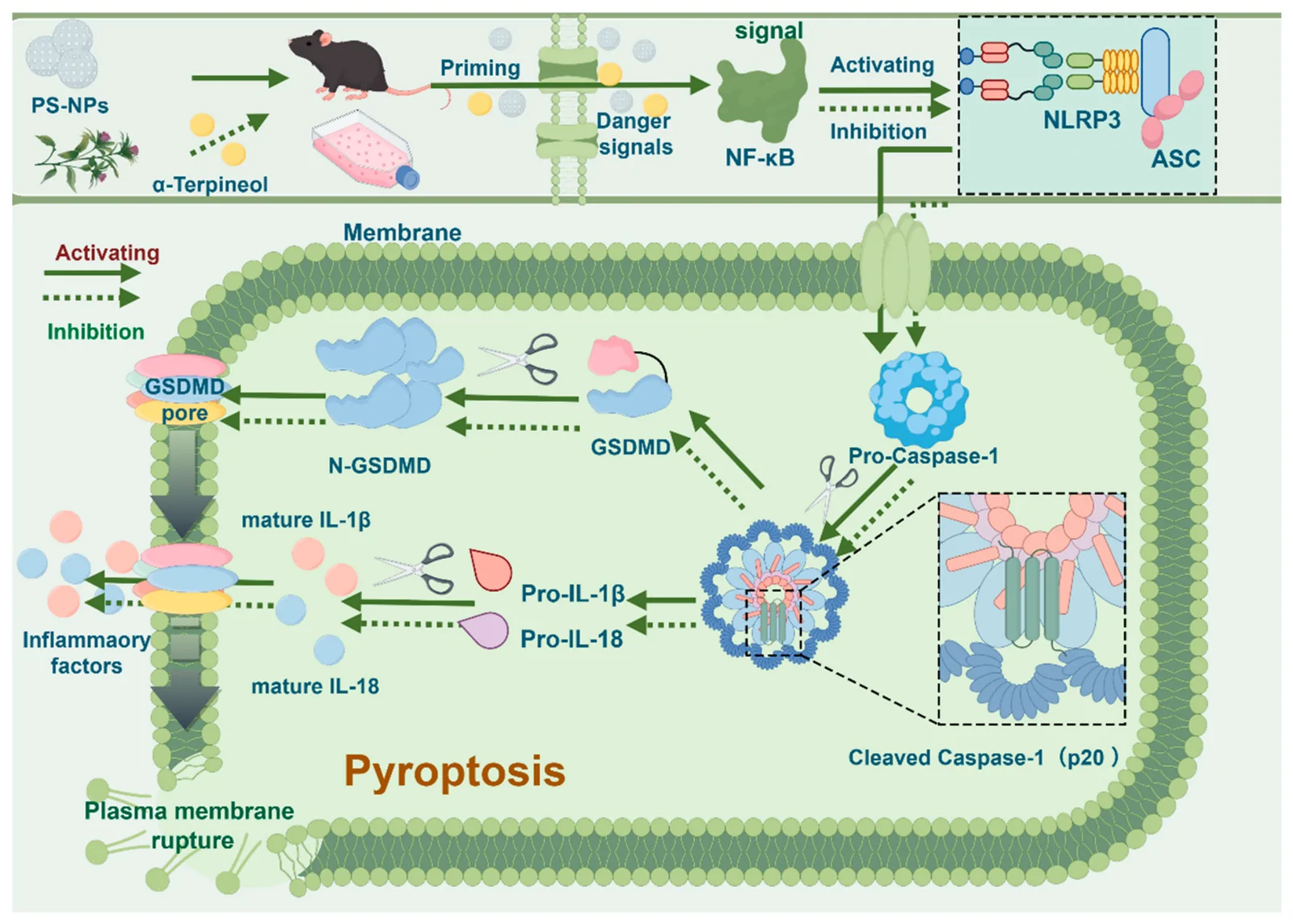
Proposed mechanism of the protective effect of α-terpineol against PS-NP-induced pulmonary injury through NF-κB-associated inflammatory signaling and GSDMD-mediated pyroptosis.

In summary, the current investigation demonstrated that exposure to PS-NPs caused tissue damage and pulmonary inflammation, along with alterations in pyroptosis linked to GSDMD and NF-κB. Exposure to PS-NPs elevated inflammatory cytokine levels and pyroptosis-related responses in A549 cells; comparable changes were noted in mouse lung tissues along with morphological and functional abnormalities to the lungs. Treatment with α-terpineol alleviated lung damage and decreased the inflammatory response and pyroptosis-related alterations caused by PS-NPs; more noticeable effects were typically seen at higher concentrations. When considered collectively, these results suggest that α-terpineol protects against pulmonary damage caused by PS-NPs, possibly through modulation of GSDMD-mediated pyroptosis and NF-κB-associated inflammatory signaling. Figure 9 shows the suggested mechanism of action.

## Figures and Tables

**Table 1:** Semiquantitative scoring criteria for histopathological assessment of lung injury.

Histopathological Parameter	Score 0	Score 1	Score 2	Score 3
**Alveolar septal thickening**	Absent	Mild	Moderate	Severe
**Inflammatory cell infiltration**	Absent	Mild	Moderate	Severe
**Alveolar edema**	Absent	Mild	Moderate	Severe
**Structural damage**	Absent	Mild	Moderate	Severe

## Data Availability

The original contributions presented in this study are included in the article/Supplementary Material. Further inquiries can be directed to the corresponding author.

## Supplementary Materials

The following are available online at https://www.mdpi.com/article/10.3390/ijms272110045/s1.
