# Supplementary material for: α-Terpineol Attenuates PS-NP-Induced Lung Injury in Association with NF-κB Signaling and GSDMD-Related Pyroptosis
Source: Int J Mol Sci. 2026 Nov 9;27(21):10045. doi: 10.3390/ijms272110045 (PMC13763748; doi:10.3390/ijms272110045)
Supplement: Supplementary file 1 [file ijms-27-10045-s001.zip › C57BL/WB C57 .pdf]

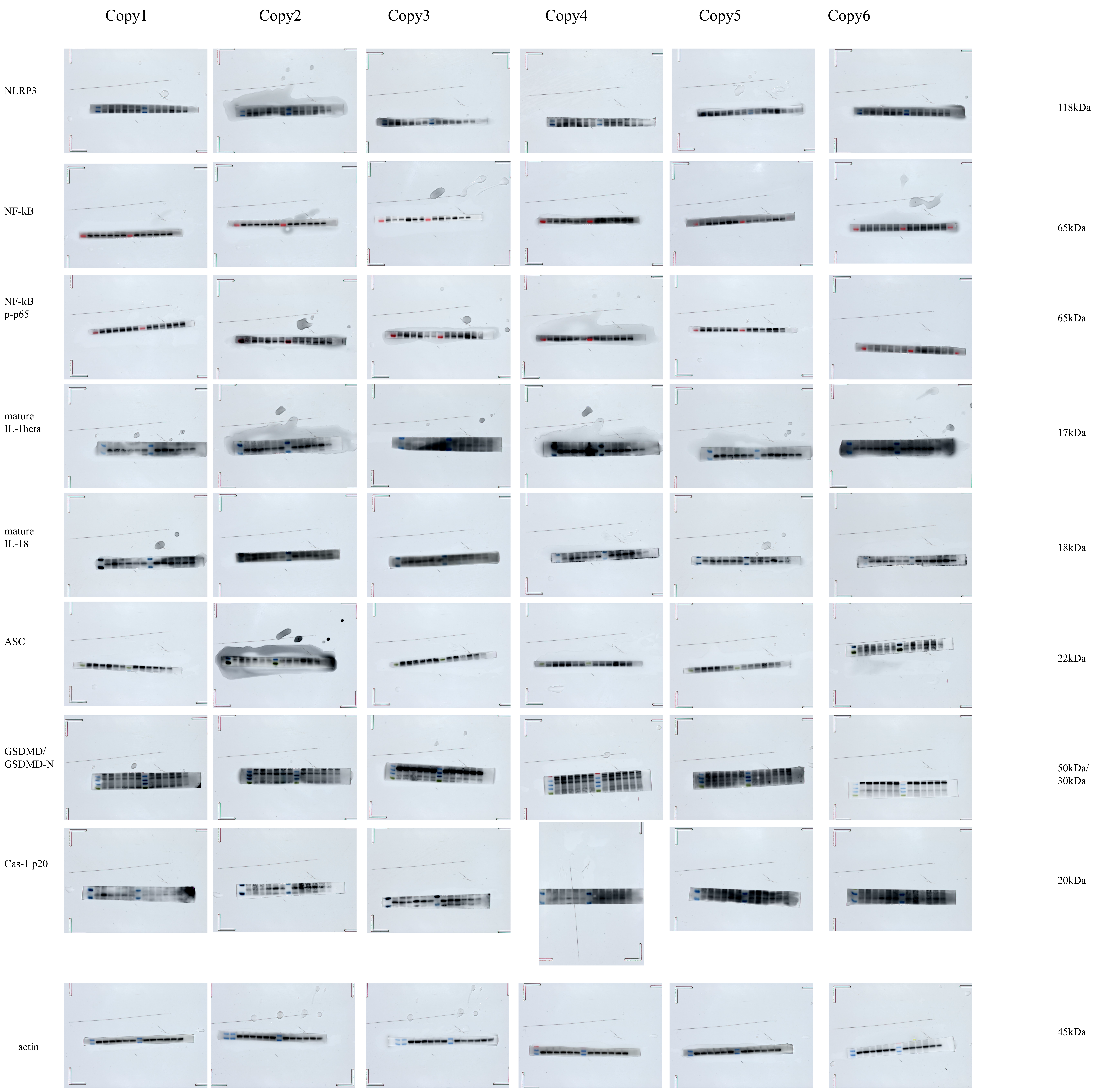

Copy1
Copy2
Copy3
Copy4
Copy5
Copy6
NLRP3
118kDa
NF-kB
65kDa
NF-kB p-p65
65kDa
mature IL-1beta
17kDa
mature IL-18
18kDa
ASC
22kDa
GSDMD/ GSDMD-N
50kDa/ 30kDa
Cas-1 p20
20kDa
actin
45kDa
